# Carotid plaque is a new risk factor for peripheral vestibular disorder: a retrospective cohort study

**DOI:** 10.1097/MD.0000000000004510

**Published:** 2016-08-07

**Authors:** Masaoki Wada, Taro Takeshima, Yosikazu Nakamura, Shoichiro Nagasaka, Toyomi Kamesaki, Eiji Kajii

**Affiliations:** a Division of Community and Family Medicine, Center for Community Medicine, Jichi Medical University, Tochigi; b Oki Clinic, Ibaraki; c Department of Public Health, Jichi Medical University, Tochigi; d Division of Diabetes, Metabolism and Endocrinology, Showa University Fujigaoka Hospital, Yokohama, Japan.

**Keywords:** atherosclerosis, carotid plaque, intima–media thickness, peripheral vestibular disorder, primary health care, retrospective cohort study

## Abstract

Many chronic diseases are associated with dizziness or vertigo, as is peripheral vestibular disorder (PVD). Although carotid plaque development is linked to atherosclerosis, it is unclear whether such plaques can lead to the development of PVD. We therefore conducted this study to investigate the presence of an association between carotid plaque and new PVD events.

In this retrospective study, we consecutively enrolled 393 patients ≥20 years old who had been treated for chronic diseases such as hypertension, dyslipidemia, and diabetes mellitus for ≥6 months at a primary care clinic (Oki Clinic, Japan) between November 2011 and March 2013. Carotid plaque presence was measured with high-resolution ultrasonography for all patients. During a 1-year follow-up period, an otorhinolaryngologist diagnosed and reported any new PVD events (the main end point). Hazard ratios (HRs) and 95% confidence intervals (CIs) for new PVD occurrence were estimated using the Cox proportional hazard regression model.

The mean age of the participants was 65.5 years; 33.8% were men, and 12.7%, 82.4%, and 93.1% had diabetes mellitus, hypertension, and dyslipidemia, respectively. There were 76 new PVD events; patients with carotid plaque had a greater risk of such events (crude HR: 3.25; 95% CI: 1.62–6.52) compared to those without carotid plaque. This risk was even higher after adjusting for traditional risk factors for atherosclerosis (adjusted HR: 4.41; 95% CI: 1.75–11.14).

Carotid plaques are associated with an increased risk of new PVD events.

## 1 Introduction

Dizziness and vertigo are the most frequently observed symptoms in many communities,^[1,2]^ and are among the chief complaints described at medical institutions.^[3–8]^ Therefore, community primary care clinics must be adept at attending to patients with dizziness and vertigo. The most frequent cause of these symptoms is peripheral vestibular disorder (PVD).^[9–11]^

Common inner ear diseases include PVD and hearing impairment. Of note, a previous study reported an association between sensorineural hearing impairment and atherosclerosis; flow-mediated dilation of the brachial artery (an early marker of atherosclerosis) was significantly lower in patients with sensorineural hearing loss than in control patients.^[12]^ Another study reported that carotid intima–media thickness (CIMT) and carotid plaques were positively associated with hearing impairment.^[13]^ On the other hand, the mechanism of the onset of dizziness/vertigo is unclear. Recently studies have shown metabolic syndrome and lifestyle-related diseases to be associated with dizziness/vertigo.^[14–18]^ Diabetes was significantly associated with vestibular dysfunction according to the National Health and Nutrition Examination Survey of 2001 to 2004.^[14]^ A previous study reported that metabolic syndrome was significantly more common among men with vertigo compared to that among men in the general adult population.^[15]^ In another study of overweight individuals (i.e., those with a body mass index [BMI] ≥25 kg/m^2^), hypertension and glucose metabolism disturbances were significantly more common among patients with vertigo than among control subjects.^[16]^ Elevated low-density lipoprotein cholesterol (LDL-C) levels and a higher prevalence of diabetes mellitus were noted among patients with vertigo, according to the findings of a previous report.^[17]^ Additionally, we reported that patients who visited our primary care clinic in Japan with dizziness/vertigo exhibited higher incidences of PVD as well as chronic diseases such as hypertension, dyslipidemia, and diabetes mellitus.^[18]^ Taken together, these reports suggest that chronic diseases are associated with dizziness/vertigo.

High-resolution carotid ultrasonography is one of the methods used to evaluate the progression of atherosclerosis associated with chronic diseases; it is a noninvasive modality that evaluates carotid arterial remodeling and carotid plaques. Carotid plaque is an established indicator of generalized atherosclerosis and an important risk factor for stroke and myocardial infarction.^[19–28]^ Asymptomatic and preclinical carotid plaque is considered a more powerful predictor of vascular events than CIMT, ankle–brachial index, and abdominal aortic diameter.^[26,29,30]^ Carotid plaque presence is a highly sensitive method for identifying subclinical vascular diseases and is considered a risk factor for cardiovascular disease.^[31]^

However, it is unclear whether carotid plaque is associated with PVD. Therefore, we conducted this study in order to determine whether an association exists between carotid plaques and new PVD events.

## 2 Methods

### 2.1 Study design

This was a retrospective cohort study.

### 2.2 Participants and setting

We consecutively enrolled 393 patients who had been treated for chronic diseases such as hypertension, dyslipidemia, and diabetes mellitus for at least 6 months at a single primary care clinic (Oki Clinic) between November 2011 and March 2013. All patients were ≥20 years old and were followed for at least 1 year, during which they were checked for any onsets of PVD. Particulars of the Oki Clinic have been described previously.^[18]^

### 2.3 Measurements at baseline

We examined age, sex, BMI (kg/m^2^), smoking status, alcohol drinking status, systolic blood pressure (mm Hg), diastolic blood pressure (mm Hg), total cholesterol (mg/dL), LDL-C (mg/dL), high-density lipoprotein cholesterol (mg/dL), triglycerides (mg/dL), glycosylated hemoglobin (HbA1c, National Glycohemoglobin Standardization Program [NGSP]; %), and serum creatinine (mg/dL). BMI was calculated as weight (kg) divided by the square of height (m). Smoking status was categorized as current, former, or never smoking; a former smoker was defined as a person who had given up smoking by the time of baseline data acquisition but had smoked previously. Alcohol drinking status was similarly classified as current, former, or never drinking. Dyslipidemia was defined as LDL-C ≥130 mg/dL, high-density lipoprotein cholesterol <40 mg/dL in men and <50 mg/dL in women, triglyceride ≥150 mg/dL, total cholesterol ≥200 mg/dL, or taking medication for dyslipidemia.^[32]^ Hypertension was defined as systolic blood pressure ≥140 mm Hg, diastolic blood pressure ≥90 mm Hg, or the use of antihypertensive medication.^[33,34]^ Diabetes mellitus was defined as fasting plasma glucose ≥126 mg/dL, nonfasting plasma glucose ≥200 mg/dL, HbA1c (NGSP) ≥6.5%, or use of antihyperglycemic medication.^[35,36]^

### 2.4 Carotid plaques

Carotid ultrasound evaluation was performed by a single trained and certified operator. All ultrasound examinations were performed using an ALOKA α-7 ultrasound instrument with a 10-MHz B-mode transducer (Hitachi Aloka Medical Corp, Tokyo, Japan). Patients were examined in a supine position with their necks extended. The patient's head position, the sonographer's position, and the scanning angles and sequence were standardized. All segments, including both sides of the common carotid artery, the carotid bifurcation, and the internal carotid artery were scanned cross-sectionally and longitudinally. Carotid plaque was defined as focal and localized increase in the intima–media thickness (IMT) to ≥1.3 mm that did not uniformly involve the entire wall of the carotid artery.^[37–39]^

### 2.5 PVD events

The primary end points of our study were new PVD events. During the 1-year follow-up period, the participants received medical consultation within a few days of any onset of dizziness or vertigo, and a board-certified otorhinolaryngologist at the Oki Clinic diagnosed and reported new PVD events according to the relevant diagnostic guidelines, taking into account medical history and clinical views. Moreover, all diagnoses of PVD were performed by a sonographer (MW) who was blinded to measurements of a patient's carotid IMT. We classified PVD according to the relevant diagnostic guidelines.^[40]^ PVD included acute peripheral vestibulopathy (APV), benign paroxysmal positional vertigo (BPPV), and Meniere disease (MD). We adopted strict diagnostic criteria for these 3 diseases as described in the following sections.

### 2.6 Clinical diagnostic criteria for APV

APV was defined as the fulfillment of all the following 3 criteria: rotatory vertigo lasting longer than 1 minute that was associated with persistent horizontal, spontaneous nystagmus; ataxia; and no sign of cochlear or central nerve involvement.^[41,42]^

### 2.7 Clinical diagnostic criteria for BPPV

BPPV was defined as the fulfillment of all of the following 4 criteria: vertigo associated with a characteristic mixed torsional and vertical nystagmus provoked by the Dix–Hallpike test, a latency (typically of 1–2 seconds) between the completion of the Dix–Hallpike test and the onset of vertigo and nystagmus, the paroxysmal nature of the provoked vertigo and nystagmus (i.e., an increase and then a decline during a period of 10–20 seconds), and fatigue (i.e., a reduction in vertigo and nystagmus when the Dix–Hallpike test was repeated).^[43,44]^

### 2.8 Clinical diagnostic criteria for MD

MD was defined as the fulfillment of all of the following 3 criteria: ≥2 definitive spontaneous episodes of vertigo lasting ≥20 minutes, audiometrically documented hearing loss on at least 1 occasion, and tinnitus or aural fullness in the treated ear.^[45,46]^

### 2.9 Analysis

All analyses were performed using Stata, version 12.1 (Stata Corp, College Station, TX). Data are presented as mean ± standard deviation and population percentages. Continuous variables were compared using *t* tests, and categorical variables were compared using χ^2^ tests. Incidence rates are presented as events per 1000 person-years. We calculated the incidence rate for our end points of new PVDs according to the presence of carotid plaque.

We used the Kaplan–Meier method to estimate the cumulative incidence of new PVDs. The Cox proportional hazard regression model was used to estimate the hazard ratios (HRs) and 95% confidence intervals (CIs) of new PVDs. For these analyses, patients were divided into 2 groups according to whether carotid plaque was present. These statistical analyses were used to examine associations between incidences of new PVDs and presence of carotid plaque by adjustment for traditional atherosclerotic risk factors (age, sex, BMI, smoking status, alcohol drinking status, systolic blood pressure, HbA1c [NGSP], and LDL-C). Statistical significance was defined as *P* < 0.05.

### 2.10 Ethical matters

This study was approved by the clinical research institutional review board of Jichi Medical University (approval number: 14-01; approval date: May 22, 2014). All participants provided verbal informed consent to participate in this study; the above-mentioned institutional review board waived the requirement of written informed consent. All the examinations and medical reviews involved in this study are regularly performed for patients with chronic diseases regardless of enrollment; hence, there are no additional efforts or risks as far as the participants were concerned. We noted participants’ consent in their medical records.

## 3 Results

Baseline characteristics of the 393 study participants are shown in Table 1. The mean age of patients at entry was 65.5 years, and 33.8% were men. Of the 393 study participants, 12.7%, 82.4%, and 93.1% had diabetes mellitus, hypertension, and dyslipidemia, respectively. Clinical and biochemical characteristics of patients based on the presence of carotid plaques are shown in Table 2. Significant differences in age, sex, and smoking status were noted between the 2 groups.

**Table 1:**
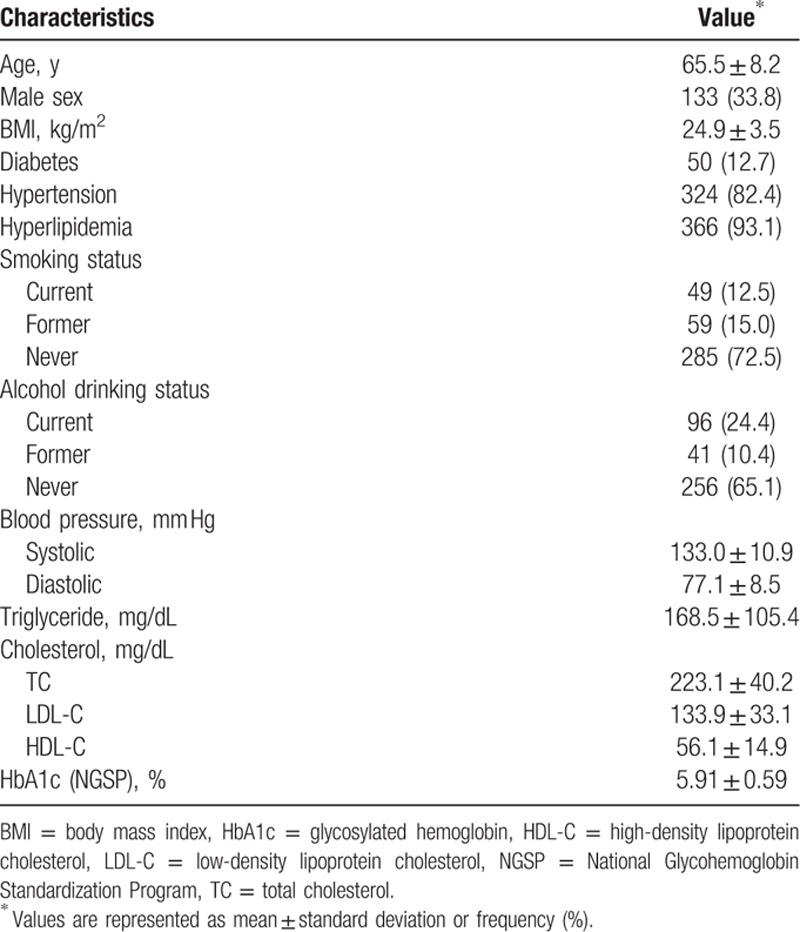
Baseline characteristics of the 393 study participants.

**Table 2:**
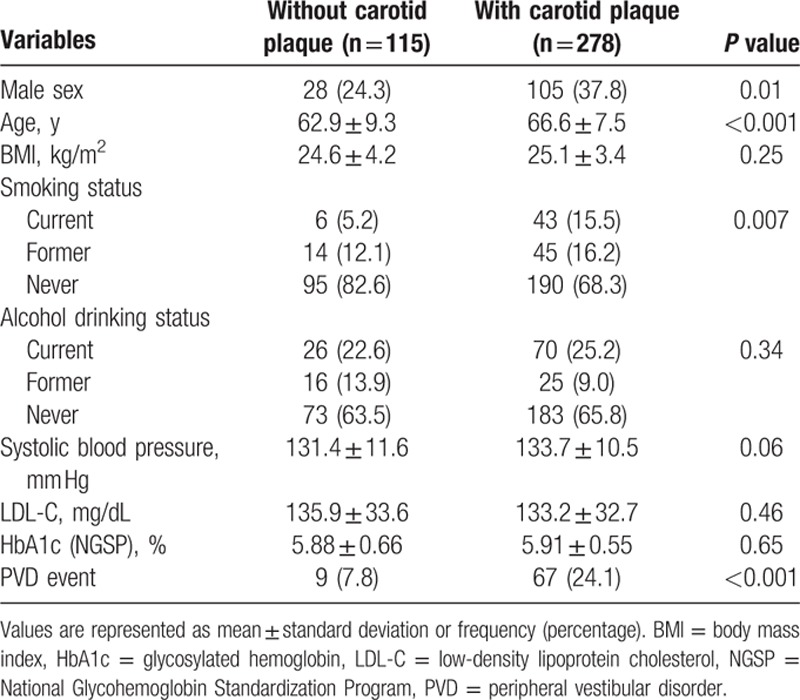
Clinical and biochemical characteristics of patients according to the presence of carotid plaque.

All 393 participants (100%) received follow-up; the median follow-up period was 633.0 days. During the 662.5 person-years of follow-up, 76 patients experienced new PVD events. Of these, 61 (80.3%) had APV, 12 (15.8%) had BPPV, and 3 (3.9%) had MD. The incidence rate for the PVD end point was 115.7 per 1000 person-years (95% CI: 92.2–142.6).

**Figure 1:**
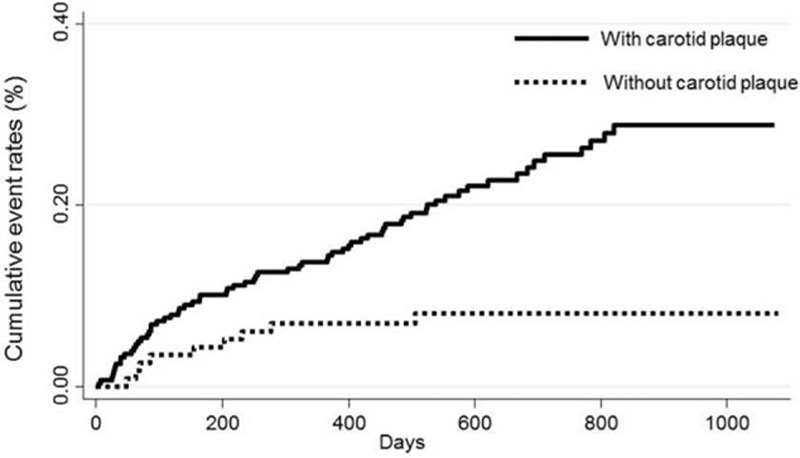
Unadjusted cumulative incidences of peripheral vestibular disorder (PVD), the end point, according to the presence of carotid plaques. Among participants with carotid plaques, there were 67 new events of PVD, whereas only 9 events occurred in those without carotid plaques.

Among the 76 participants who experienced new PVDs, 67 had carotid plaque. There were only 9 new PVD events among those without carotid plaque. The incidence rate of new PVDs was 145.7 per 1000 person-years (95% CI: 114.7–181.2) among patients with carotid plaques and 44.1 per 1000 person-years (95% CI: 20.4–82.1) among those without. Figure 1 shows the cumulative incidence of new PVDs according to the presence or absence of carotid plaque.

The crude HR for new PVD events for participants with carotid plaque (using participants without plaque as the reference) was 3.26 (95% CI: 1.62–6.58). On adjusting for age, sex, BMI, smoking status, alcohol drinking status, systolic blood pressure, serum LDL-C, and HbA1c (NGSP), the HR was 4.41 (95% CI: 1.75–11.14) when analyzing the entire cohort (n = 349; Table 3).

**Table 3:**
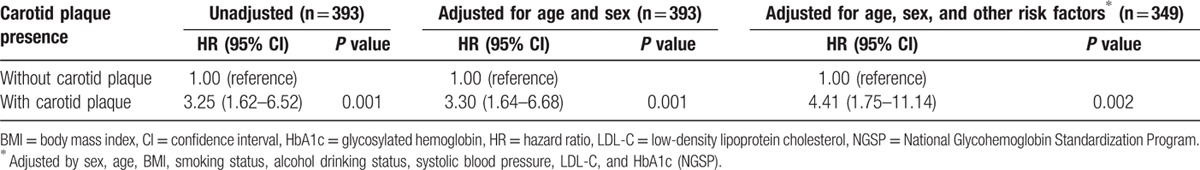
Cox proportional hazard analyses of peripheral vestibular disorder end points according to carotid plaque presence.

## 4 Discussion

To the best of our knowledge, this is the first study aimed at investigating whether a link exists between new PVD events and carotid plaque. Indeed, we found a significant association between these phenomena, and revealed that the presence of carotid plaque was a risk factor for new PVD events in primary care patients with chronic diseases such as hypertension, dyslipidemia, and diabetes mellitus.

Carotid plaque is an important risk factor for cardiovascular and cerebrovascular diseases, but has not been regarded as a predictor of PVD. In fact, we were unable to locate any precedent studies on the carotid plaque and PVD. Previous studies did report that ultrasonographic assessment of carotid plaque had a higher diagnostic accuracy than CIMT for the prediction of future ischemic strokes^[24]^ and coronary artery disease events.^[26,30]^ The High Risk Plaque BioImage study in 2012 reported that carotid plaque burden was found to correlate more strongly with subclinical early atherosclerosis events, such as subclinical coronary calcification, than CIMT.^[31]^ Carotid plaque presence may also be a suitable measure for evaluating early arteriosclerosis in the whole body. Because we considered carotid plaque a measure of early atherosclerosis in our study, this led us to conclude that atherosclerosis itself may be associated with the risk of new PVD.

Of note, ease of measurement and low cost are important considerations when determining the presence of carotid plaque. Compared with measuring IMT, no sophisticated equipment or software is required for determining carotid plaque presence. The Rotterdam study of 2004 reported that the relatively crude measures used to directly assess plaque in the carotid artery predict myocardial infarction as reliably as measuring carotid IMT, which requires more onerous techniques.^[29]^

This study suggests the possibility that atherosclerosis, endothelial dysfunction, flow instability, and hemodynamic stress in brain-feeding arteries are a cause not only of myocardial infarction and stroke but also of PVD onset. Previous studies have shown that there is enhanced expression of vascular cell adhesion molecule-1,^[47,48]^ intercellular adhesion molecule-1,^[47,48]^ and plasminogen activator inhibitor-1^[49,50]^ in the carotid atherosclerotic plaque, as well as decreased production or activity of nitric oxide.^[51]^ Furthermore, another study showed that plaque-influenced hemodynamic factors (e.g., oscillatory wall shear stress) restricted endothelial function owing to impaired nitric oxide–mediated vasodilation in pig carotid arteries.^[52]^ Another study revealed that vulnerability upstream of the plaque region is associated with enhanced neovascularization, hemorrhage, and fibrous cap thinning that lead to plaque progression and rupture.^[53]^ These changes that manifest with atherosclerosis may promote plaque formation as well as the development of intravascular thrombosis and impaired vasodilation in human carotid arteries. Such changes, which cause transient bloodstream disorders and dysfunction of the inner ear, may lead to new PVD events. Pathologic elucidation of the mechanisms of new PVD onset will be necessary in future studies.

### 4.1 Strengths and limitations

This study has 3 notable strengths. First, the participants who complained of dizziness/vertigo were examined by a board-certified otorhinolaryngologist; therefore, PVD was detected more accurately. Second, we followed up with all the participants; this led to a more accurate representation of the prevalence of dizziness/vertigo and PVD. Third, all carotid ultrasound measurements were performed by an experienced operator who was trained by the Department of Clinical Laboratory Medicine at Jichi Medical University. Therefore, carotid plaque was measured more accurately.

On the other hand, the study has several limitations as well. First, BMI values of 44 patients (11.2%) were unavailable. In fact, approximately 10% of the data was missing; this may have introduced bias into our findings. Second, our statistical analysis was adjusted for several known confounding factors; however, we were unable to account for yet unknown factors. Third, our investigation was limited by the study's selection bias; because the present study was conducted at a single primary care clinic, the external validity of our findings ought to be evaluated in patients at multiple medical institutions.

In conclusion, we revealed that the presence of plaque in the carotid artery is associated with the occurrence of new PVD events. However, our study had several limitations (unavailable data points, potential unknown confounders, and bias inherent in a single institution study). Therefore, further studies are required to determine whether our results are representative of patients in the general population at multiple medical institutions.
